# Inferring the connectivity of coupled oscillators from time-series statistical similarity analysis

**DOI:** 10.1038/srep10829

**Published:** 2015-06-04

**Authors:** Giulio Tirabassi, Ricardo Sevilla-Escoboza, Javier M. Buldú, Cristina Masoller

**Affiliations:** 1 Departament de Fisica i Enginyeria Nuclear, Universitat Politécnica de Catalunya, 08222 Terrassa, Barcelona, Spain; 2 Center for Biomedical Technology, Technical University of Madrid, Pozuelo de Alarcón, 28223 Madrid, Spain; 3 Centro Universitario de los Lagos, Universidad de Guadalajara, Lagos de Moreno, Jalisco 47460, Mexico; 4 Complex Systems Group, Universidad Rey Juan Carlos, 28933 Móstoles, Madrid, Spain

## Abstract

A system composed by interacting dynamical elements can be represented by a network, where the nodes represent the elements that constitute the system, and the links account for their interactions, which arise due to a variety of mechanisms, and which are often unknown. A popular method for inferring the system connectivity (i.e., the set of links among pairs of nodes) is by performing a statistical similarity analysis of the time-series collected from the dynamics of the nodes. Here, by considering two systems of coupled oscillators (Kuramoto phase oscillators and Rössler chaotic electronic oscillators) with known and controllable coupling conditions, we aim at testing the performance of this inference method, by using linear and non linear statistical similarity measures. We find that, under adequate conditions, the network links can be perfectly inferred, i.e., no mistakes are made regarding the presence or absence of links. These conditions for perfect inference require: i) an appropriated choice of the observed variable to be analysed, ii) an appropriated interaction strength, and iii) an adequate thresholding of the similarity matrix. For the dynamical units considered here we find that the linear statistical similarity measure performs, in general, better than the non-linear ones.

Systems composed by interacting dynamical elements are ubiquitous in nature. In many situations, it is desirable to model such systems as networks of coupled oscillators, where the nodes represent the individual units and the links represent the interactions among them. These interactions, which arise due to a variety of physical mechanisms, are often unknown, or only partially understood, and thus, a complete knowledge of the network topology is lacking. Technical limitations, or the nature of the network itself, make sometimes impossible to infer how the nodes are linked among each other. Within this framework, it is important to address the problem of how to optimally infer the connectivity of a system from the observation of the dynamics of its interacting elements^1^,^2^,^3^,^4^,^5^,^6^,^7^,^8^. Two paradigmatic examples of this situation are brain functional networks and climate networks^9^,^10^,^11^,^12^,^13^,^14^,^15^,^16^.

In these cases, networks are built through the statistical study of the correlations between the time series associated to different physical regions (areas of the brain or geographical areas of Earth), which, in turn, are the nodes of the network. First, a covariance matrix representing the coordinated activity between all pairs of nodes is calculated, and then, a functional network is obtained by thresholding the covariance matrix: if two nodes display a high statistical similarity they are considered linked, otherwise not^13^,^17^. In this way, from the filtered covariance matrix an adjacency matrix is obtained. In the following, we will refer to this inferred matrix as the functional network associated with the system. The functional network does not necessarily correspond to the real physical connectivity: In fact, two or more nodes are influenced by the same noise source, or some common external forcing, their dynamics might result highly correlated even if they are not directly interacting.

However, as one aims at finding a functional network that resembles as close as possible the physical connectivity of the system, it is crucial to develop reliable techniques capable of unveiling interdependencies, based on the observation of the dynamics of the units that compose the system.

In the recent years, numerous approaches of network inference have been proposed^3^,^4^,^5^,^6^,^7^,^18^,^19^. For example, one method was based on studying the response of coupled oscillators when they are perturbed by an external forcing^3^; in this case the network was inferred after a sufficiently large amount of tests with different driving intensities. In^6^ the functional network was retrieved by using a delayed feedback control that drives the network into steady states. Other methods rely on the direct observation of the nodes’ dynamics. For example, in^5^ and^8^, the Laplacian matrix is reconstructed using the covariance matrix of the nodes’ time-series, and from the properties of the Laplacian, the links are inferred.

In^7^ a system composed by discrete-time units was studied (various maps were considered, including the Logistic and Circle maps) and it was found that (i) under appropriated coupling conditions the network can be perfectly inferred, these conditions being a weak coupling regime where the network is neither fully synchronized, nor completely desynchronized and (ii) regarding the statistical similarity measure used for inferring the system structural connectivity, the mutual information in general outperforms the cross-correlation.

Here we test these observations by considering a set of phase oscillators and a set of chaotic three-dimensional oscillators. We chose the Kuramoto system because it is a paradigmatic model of many physical, biological and chemical systems^20^. To test the inference method under controllable experimental conditions, we also consider Rössler oscillators, operating in the chaotic regime, which are implemented electronically. I the case of the Rössler oscillators we will use only one component of the three-dimensional time-series of the oscillators instead of using a multivariate analysis. In this way we can test what happen with those systems in which only a projection of a multidimensional dynamics is available for being monitored, as in brain and climate network.

To the best of our knowledge, no previous work in the literature has performed a detailed comparison between these different variables and different statistical measures applied to the structural network reconstruction. Our study is certainly of interest because it allows to understand the advantages and drawbacks of using each of the methods, and their dependence on the coupling strength of the interacting units. Moreover, to perform our analysis, we not only use synthetic data generated via model simulations, but also test the methods with experimental data recorded from a real system of interacting electronic circuits.

We show that the inference method performs well for both, Kuramoto phase oscillators and Rössler chaotic oscillators. In particular we find that, contrarily to what was found in^7^ for discrete-time maps, in the Kuramoto oscillators’ case the cross correlation is usually the best performing similarity measure. We also show that the inference method is able to reconstruct the physical connectivity of the system (in the following, referred to as the structural network) for a broad range of parameters, even when the oscillators are synchronized. This is due to the presence of independent noise sources in the individual oscillators, which prevent them to assume identical states.

## Methods

In this section we first describe the two systems of coupled oscillators analysed: Kuramoto phase oscillators and Rössler chaotic oscillators, which were implemented experimentally via electronic circuits. Then, we describe the statistical similarity measures (SSMs) used to infer the system’s physical connectivity: the absolute value of the cross-correlation and the mutual information. The latter was computed in two ways: i) via the usual estimation of probabilities from histogram of time-series values and ii) via the probabilities of symbolic patterns, using the ordinal transformation of time-series. We will explain these quantities in detail in the following sub-sections.

### Kuramoto Phase Oscillators

The rate equations for *N* coupled Kuramoto phase oscillators are^21^.
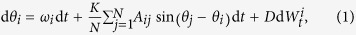
where *θ*_*i*_ and *ω*_*i*_ are respectively the phase and the natural frequency of the oscillator _*i*_, and *k* is the coupling constant between the oscillators. *dW*_*t*_^*i*^ is a Weiner process having 0 mean and variance tuned by the parameter 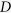. The matrix **A**, usually referred to as the adjacency matrix, defines the structural network; it indicates the physical connectivity of the system, i.e., the existing interactions among pairs of nodes. In detail: *A*_*ij*_ = 1 if two oscillators are linked and *A*_*ij*_ = 0 otherwise. We consider symmetric bidirectional coupling, thus **A** is a symmetric matrix.

The distribution of the natural frequencies is commonly considered to be centered in 0, since the equations are invariant under a phase translation, 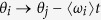. Moreover, the distribution is often taken symmetric^20^, so we assume here that the oscillators’ frequencies are uniformly distributed in the interval [−Ω,Ω].

The collective dynamics of the oscillators is usually monitored by means of a complex order parameter, *R*, defined as^20^,^28^.
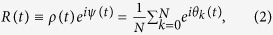
where *ψ*(t) represents the average phase over the ensemble of oscillators and *ρ*(t) quantifies the degree of synchronisation, ranging from 0 (no synchronisation) to 1 (perfect synchronisation).

From the oscillator phases, *θ*_*i*_(t), we derive another two quantities: i) the instantaneous frequencies,
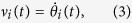


and ii) the Y-projections.
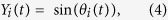
i.e., the vertical projection of the unitary vector associated to the phase *θ*_*i*_. In this way, despite the interaction involves only the oscillator phases, we have three different magnitudes to infer the underlying links: the phases, the frequencies and the Y-projections. We follow this procedure because in real-world systems it is frequent to use proxies of the quantities of interest.

### Electronic Rössler Oscillators

As stated in the Introduction, we test the network inference method with experimental data by constructing a set of coupled electronic oscillators. Specifically, the experiment consists of 12 identical piecewise Rössler electronic circuits^22^,^23^,^24^ whose dynamics can by modelled by:
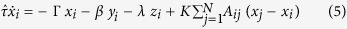

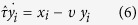

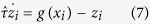
where g(x) is the piecewise linear function
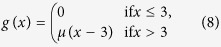


*x*_*i*_, *y*_*i*_ and *z*_*i*_ are the oscillator state variables and **A{*****A***_*ij*_} is the structural connectivity matrix. We consider a symmetric bidirectional coupling, thus **A** being a symmetric matrix.

**Figure 1:**
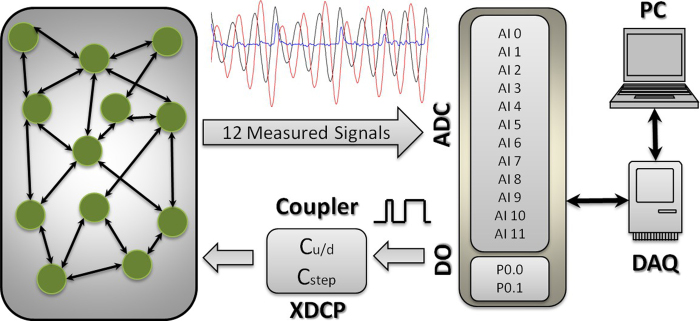
Diagram of the experimental setup representing a network of 12 electronic Rössler oscillators. The bidirectional coupling is adjusted by means of 12 digital potentiometers X9C104 whose parameters *C*_*u/d*_ (control of increment/decrement of the resistance) and *C*_*step*_ (time-step to change the value of the resistance) are controlled by a digital signal via a DAQ Card. The output of each circuit is sent to a voltage follower that acts as a buffer and, then, sent to the analog ports (AI 0; AI 1; AI 11) of the same DAQ Card. The whole experiment is controlled from a PC with a Labview Software.

Figure 1 displays a schematic diagram of the experimental setup. The parameters of the individual circuits are such that their dynamics is chaotic, and the coupling parameter is varied in a range such that the system is not synchronized. The full details of the experiment are presented in the Supplementary Material.

Since the most common scenario in real systems is not having a complete knowledge of the dynamical state of the system and, typically, not all relevant variables can be observed, we only took into account one of the three variables of the oscillators, *x*(t), where the interaction actually takes place, and obtained an univariate time-series for each oscillator. Next, from the time-series of *x*_*i*_(t) we obtained the time-series of phases, *θ*_*i*_(*t*), through a Hilbert transformation, and from the phases we computed the instantaneous frequencies, *v*_*i*_(*t*). In this way, also for Rössler oscillators we have three magnitudes from which we can attempt to infer the underlying links: *x*, *θ* and *v*.

### Statistical similarity measures

The construction of the functional network is based on evaluating the similarity of the dynamics of the oscillators through the computation of a statistical similarity measure (SSM). In this work we used three SSMs, namely the absolute value of the cross correlation (also known as Pearson’s coefficient) CC, the mutual information MI and the mutual information of the time series ordinal patterns MIOP^25^. The former is a linear measure and the two latter are non-linear ones.

Before computing these SSM values, every time-series are normalized to have zero mean and unitary variance. In addition, for the time-series of the oscillator phases we remove the linear trend.

CC measures the linear interdependence between the dynamics of two nodes of the network. Being *a*_*i*_(*t*) and *a*_*j*_(*t*) the time series in nodes *i* and *j*, normalized to have unitary variance, the Pearson’s coefficient is
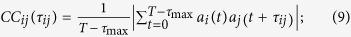
where *T* is the length of the time-series, τ_*ij*_ is a lag-time between them and τ_max_ is the maximum lag considered.

On the other hand, the mutual information is:
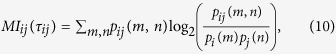
where *p*_*i*_ is the probability distribution associated to *a*_*i*_(*t*), *p*_*j*_ is the one associated to a_*j*_(*t*+τ_*ij*_), and *p*_*ij*_ is the joint probability distribution of *a*_*i*_(*t*), a_*j*_(*t*+τ_*ij*_), estimated by means of time-series of *T* − *τ*_max_ points.

The MIOP is a variant of MI, that is computed from ordinal patterns associated with the time series. In the ordinal transformation every time-series is transformed into a sequence of symbols, referred to as *ordinal patterns*. With this approach, the time series is divided into segments of length *Q*, then each value within the segment is assigned a symbol according to the ranking of the values inside each segment. The ordinal patterns take into account the local variations of the amplitude of the signal rather than the precise value^25^.

For example, if *Q* *=* 3 and *a*(*t*_*i*_) > *a*(*t*_*i*+1_) < *a*(*t*_*i*+2_), the *a*(*t*_*i*_) value is replaced by the ordinal pattern “012” while if *a*(*t*_*i*_) > *a*(*t*_*i*+1_) < *a*(*t*_*i*+2_) the pattern “210” is used instead, and so forth. Considering patterns of length *Q* there are *Q*! different possible patterns.

In this way the time series are transformed into sequences of symbols, and the MI is computed from Eq. (10), with *p*_*i*_, *p*_*j*_ and *p*_*ij*_ being the probabilities of the possible symbols. A notable characteristic of this method is that it is very robust to noise^25^ and able to capture different time-scales^15^.

In the following, for computing the MI measure, the probability distributions of values will be estimated via histograms with a number of bins given as *N*_*bin*_ ∼ *O*(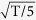 where *T* is the length of the time-series. This allows for the robust estimation of the joint probabilities. The MIOP measure was computed with ordinal patterns of *Q* *=* 4. Using larger *Q* values requires extremely long time series, since the number of possible ordinal patterns increases as *Q*!. In real systems, the length of observed time series is normally limited, and thus we restricted our simulations to the same constraints in order to obtain results that can be exported to real systems.

In order to reduce the effect of noise, the SSM values were averaged over 10 windows: each time series was divided in 10 non-overlapping sections each of length *T* and in each section the SSM was computed as a function of the lag time τ_*ij*_. Then, the statistical similarity measure is defined as the maximum value with respect to the lag τ_*ij*_,
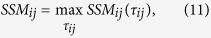


being *SSM* either *CC*, *MI* or *MIOP*.

We use the maximum SSM value to allow for the existence of lag-time between the time series. While we could have estimated the SSM via a moment of the SSM(τ_*ij*_) distribution (e.g., the mean value plus a certain number of standard deviations) we chose this approach because it has been used to infer functional climate networks (see, e.g. 26,). The lag time distribution in our case is usually peaked around 0 (not shown).

When computing the SSM, the maximum value can depend significantly on the interval of values allowed for τ_ij_^27^. Here we consider τ_max_ = T/5. Since lagging two time series reduces the number of available data to compute the SSM value, τ_max_ was chosen as a compromise, in order to allow for sufficiently long lag-times while simultaneously keeping enough data to evaluate reliably the SSMs.

### Network reconstruction

The computation of the *SSM*_*ij*_ between all pair of nodes leads to a *N* × *N* matrix from which the structural links can try to be inferred by a suitable thresholding of the matrix. In order to chose an adequate threshold, we compute the mean value of the matrix elements, *M*_*s*_, and their associated standard deviation, σ as
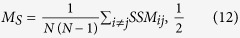
and
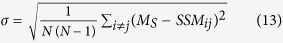
With these two quantities we define the threshold *TH* as
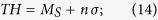
where *n* is a number that was heuristically tuned to obtain the best possible reconstruction, that is to obtain values of inferred links as high as possible keeping low the values of mistakes. With this threshold we prune the **SSM** matrix to reconstruct the network topology in the following way:
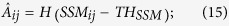
where **Â** denotes the reconstructed adjacency matrix and *H* is the Heavyside step-function. The matrix **Â** is the inferred network, which is usually referred to as the functional network. Here we aim at analysing up to which extent the functional connectivity (represented by **Â**) resembles the physical connectivity (represented by the adjacency matrix, **A**). The strong hypothesis that we make here is that high values of SSM correspond to structural couplings. Of course this may be not always the case. For example, a high synchronisation among the oscillators will imply high values of correlations, regardless whether they are directly coupled or not. As another example, oscillators that are under common external forcing will have correlated dynamics, and thus they will show high degree of statistical similarity, even if there are no structural connections among them.

## Results

To test the inference method described in the previous section, we consider a structural network **A** that is a fully random (Erdös-Rényi) network. In such a network the nodes are connected with a probability *p*, and the link density is equal to *p*.

We highlight at this point that the model presents five parameters: the number of oscillators, *N*, the coupling constant, *K*, the network density, *p*, the noise intensity *D*, and the width of the distribution of natural frequencies, Ω. In the simulations, unless otherwise specifically stated, *N* = 12, *P* = 0.27, *D* = 0.05 and Ω = 20*p/N*. These values of *N* and *p* are chosen to fit the experiment with Rössler oscillators. Moreover, the value of *p* is large enough that, for *N* = 12,there are few disconnected nodes. The issue of having *few* disconnected nodes is not a problem, because we are also interested in checking if we are able to detect them (and in fact we do, as we show below we achieve perfect reconstruction of the Kuramoto network applying the method to the oscillators’ frequencies).

The only free parameter left is the coupling strength *K*. For each value of *K*, we studied the system’s dynamics by performing 100 stochastic simulations with different values of the oscillators’ frequencies, network realizations, noise realizations, and initial conditions. The total simulation time was 2 × 10^3^*N/p*; the first half of the simulation was disregarded as a transient time, while the second half was used for the calculations. The data obtained in the second half was subsequently coarse-grained to mimic a measurement process that results in time-series of 10^4^ data points which were divided in 10 non overlapping sectors of *T* = 10^3^ data points.

Varying *K* we studied the performance of the reconstruction method in the nine cases that result from the combination of the three SSMs (CC, MI, MIOP) computed from the three time-series in each node: phases, frequencies and 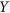-projections (Kuramoto) or *x*_*i*_ variable (Rössler).

We expect the performance of the algorithm to be significantly affected by the value of the coupling parameter K^7^. On the one hand, sufficiently low values of 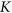 will lead the system to behave as an ensemble of uncoupled oscillators. On the other hand, when *K* is large enough the system will synchronize and the trajectories will not be distinguished leading to an all-to-all fully connected matrix. In both limits, the functional network will not recover the organization of the structural connections. Thus, it is for intermediate values of *K* where the SSM matrix can be expected to contain information about the physical connectivity of the system.

Before presenting the analysis of the network reconstruction, it is relevant to discuss the behaviour of uncoupled (*K* = 0 ) Kuramoto oscillators in the presence of Gaussian noise.

### Stochastic Uncoupled Kuramoto Oscillators

**Figure 2:**
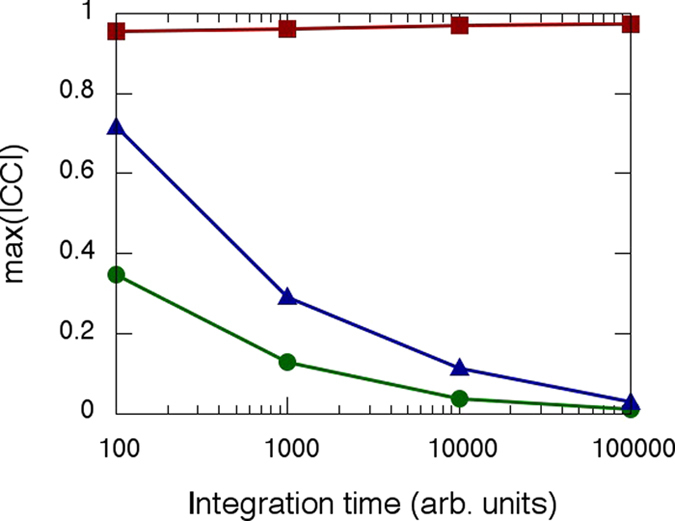
Maximum cross-correlation values between any two pair of uncoupled Kuramoto oscillators (K = 0). We analyze 120 stochastic simulation of (1) and compute max(**|**CC**|**) as a function of the number of integration steps. We plot the values obtained from the analysis of the time series of: phases *θ* (red squares), instantaneous frequencies *v* *=* *θ* (green circles), and vertical projections, *Y* *=* *sin(θ)* (blue triangles).

With *K* = 0 the dynamics of the nodes are independent Brownian processes with different trends but with the same noise strength. As it can be seen in Fig. 2, in this limit it is possible to obtain high values of CC between the time series of the oscillators’ phases, despite the fact that the oscillators are uncoupled. From Fig. 2, it is also evident that the maximum CC value is independent of the integration time. It is also independent of the noise strength, because the cross correlation is normalized with respect to the variance of the time-series, that is, the intensity of the noise. An explanation of this behaviour is provided in the Supplementary Material. Thus high correlation values between the phases of two nodes, even with a large amount of data, cannot be regarded as a reliable signature of structural interactions.

For the other two observables, the instantaneous frequencies *v* and the *Y*-projections, instead, high correlations vanish as the length of the time-series increases.

### Stochastic Coupled Kuramoto Oscillators

When switching on the interactions between the nodes, i.e., when using *K* *>* *0*, we can try to infer the structural network via the thresholding of the SSM matrices.

**Figure 3:**
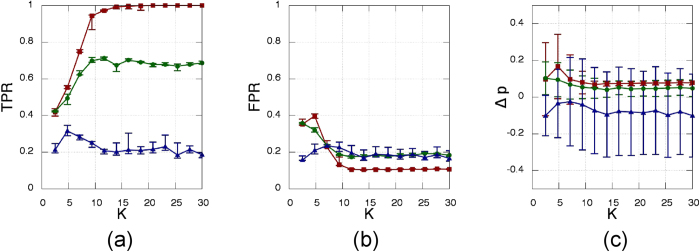
Performance of the inference algorithm for the three similarity measures considered (CC based quantities are depicted in red squares, MI in green circles and MIOP in blue triangles), applied tp the phase time-series, *θ*_*i*_. (**a**) Fraction of true positives, (**b**) fraction of false negatives, and (**c**) difference between the inferred density and the actual density of the network, Δ_*p*_, as a function of the coupling strength, *K*. Error bars are computed as the left and right standard deviation of the values obtained from over 100 stochastic simulations with different noise realisations, initial conditions, structural network realisations and natural frequencies.

Figure 3 displays the results, when the phase *θ* is the observed variable. We evaluated the performance of the method as a function of *K* by plotting the fraction of correctly inferred links (true positive ratio, TPR), the fraction of wrongly inferred links that are not present in the structural network (false positive ratio, FPR), and the difference between the density of the reconstructed functional network and the density of the structural network. Each value is the average performed over 100 stochastic simulations, and the error bars are computed as left and right standard deviations respect to the mean.

The *K* range has been chosen in order to cover the transition from non synchronisation to almost full synchronisation.

**Figure 4:**
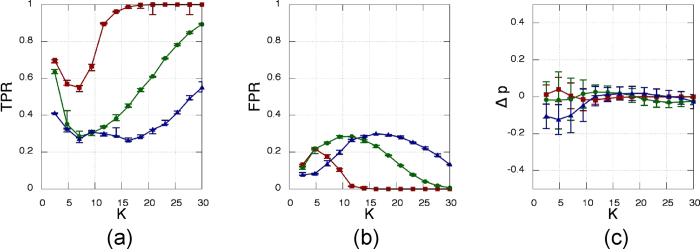
Same as in Fig. 3 but the statistical similarity measures are applied to time series of the instantaneous frequencies, ***v***_**i**_.

Figure 4 presents the results obtained from the analysis of the instantaneous frequencies, *v*_*i*_. In this case the three SSMs present a critical *K* above which they are able to infer correctly the structural network, with the fraction true positives reaching one and the fraction of false negatives being zero, both of them with null dispersion over many stochastic simulations. However, CC still reveals to be the best SSM, since it has a lower critical *K* together with both higher values of TPR and lower values of FPR. Most importantly, we note that, in spite of the fact that the oscillators interact through their relative phases (and not their frequencies), the network recovery can be indeed perfect.

**Figure 5:**
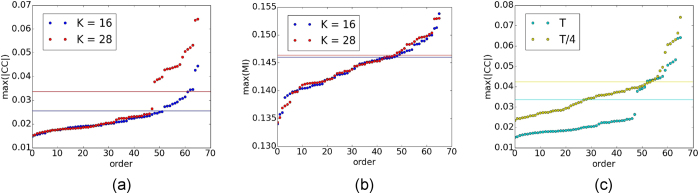
(**a**): CC values calculated from the frequencies of the Kuramoto oscillators for one realisation of the dynamics with coupling strength *K* = 16 (blue) and *K* = 28 (red). The values are ordered from the smallest to the biggest. The threshold used for computing the functional connectivity is reported as a horizontal line of the same colour of the associated CC values. As it can be seen in the case of high *K* (*i. e.* in the case of perfect reconstruction) the CC develops a gap in the middle of which is placed the threshold. **(b)**: same as (**a**) but for MI values. No gap is present now. In this case the network reconstruction is worse than in the CC case. **(c)**: same as in (**a**) for *K* = 28. In green the original time-series are used, while in yellow the sime series are just 1/4 than the original ones. Also in this case tha gap disappears, and the network reconstrucion get worse. These results are in agreement with^7^.

In Fig. 5(a) we show the values of the CC matrix in increasing order for one realisation of the frequencies of Kuramoto dynamics. We report the values for two different *K* values, one for which we have poor reconstruction (lower) and one for which have perfect reconstruction (higher). We also report the associated threshold with the same color. As it can be seen, in the case of perfect reconstruction (high *K*), the CC values develop a gap that is not present in the poor reconstruction (low *K*) case. The fact that the threshold for the high *K* is placed just in the middle of the gap show that this abrupt change in the CC values separates links from non-links. This behaviour is consistent with what found in Ref. 7. In 4(b) and (c) we show how this gap disappears if the MI is used instead of CC or if the length of the series is reduced, all cases of non-optimal reconstruction of the network.

**Figure 6:**
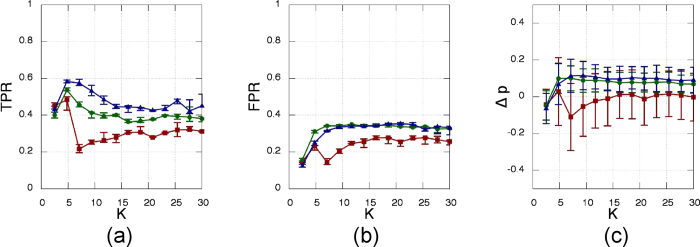
Same as in Fig. 3 but for ***Y***_***i***_ time-series.

In Fig. 6 we test the inference method with the other indirect variable, the *Y*-projection. In this case the method fails almost completely, being unable to efficiently reconstruct the structural network. However the inferred density is often consistent with that of the target network – although a certain dispersion in these values is present. Table 2 summarizes the best performance for all the possible combinations of SSMs and observables.

It is important to note that the presence of noise is fundamental, preventing the complete synchronisation of the oscillators that would make the trajectories indistinguishable. Thus, even with a relatively high value of the synchronisation parameter *r*, we can still achieve perfect reconstruction.

**Figure 7:**
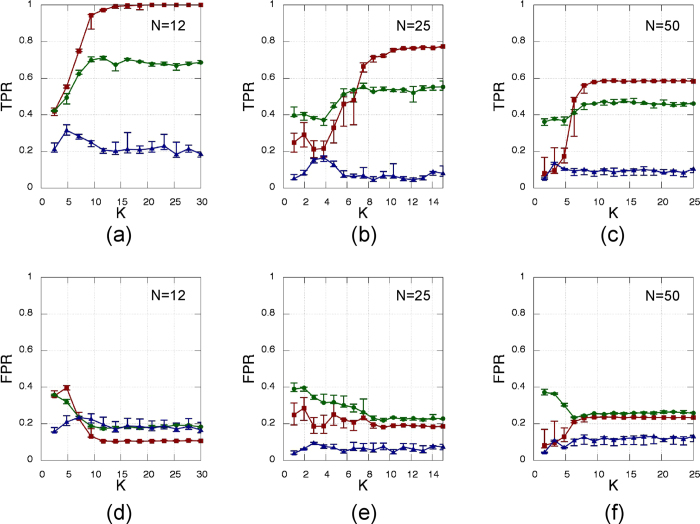
Same as Fig. 3 but for three different network sizes. The left column, **(a,d)**, is computed using the same amount of oscillators as in Fig. 3, while the other two columns refer to higher network dimensions, namely 25 oscillators **(b,e)** and 50 oscillators **(c,f)**.

Figure 7 displays the influence of increasing the system’s size, *N*, while keeping the link density constant (*p* = 0.27). As it can be seen, the only SSM showing a certain degree of resilience to the network size is the cross-correlation, which keeps decent values of TPR and FPR also for higher *N*.

**Figure 8:**
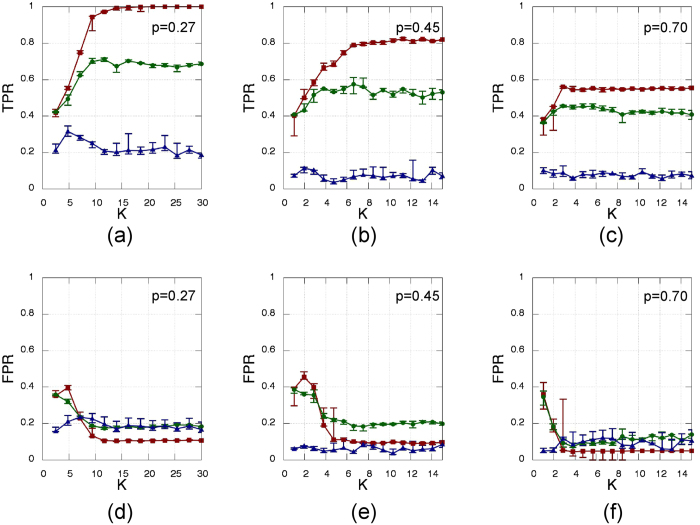
Same as Fig. 3 but for three different network densities. The left column, **(a,d)**, is computed using the same amount of links as in Fig. 3, while the other two columns refer to higher network connctivity, namely *p* = 0.45 **(b,e)** and *p* = 0.70 **(c,f)**.

In Fig. 8 we report effect of varying the network density while keeping constant the network size (*N* = 12). As it an be seen from the figure, the quality of the reconstruction decreases when increasing the network connectivity.[Table t3]

### Experimental electronic Rössler oscillators

**Figure 9:**
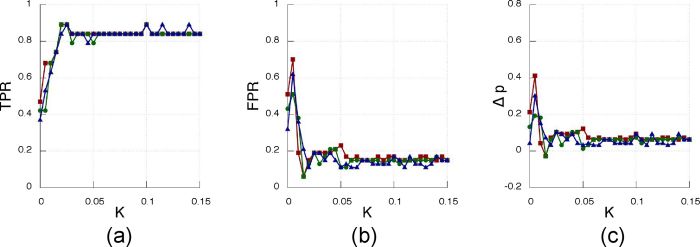
Performance of the inference algorithm for experimental data corresponding to a random network of 12 Rössler chaotic electronic circuits, for the three SSMs (CC based quantities are depicted in red squares, MI in green circles and MIOP in blue triangles). Time series contain the evolution of the *x* variable. (**a**) fraction of true positives, (**b**) fraction of false positives, (**c**) difference between the inferred density and the actual density of the network, Δ*p*, as **a** function of the coupling strength K.

**Figure 10:**
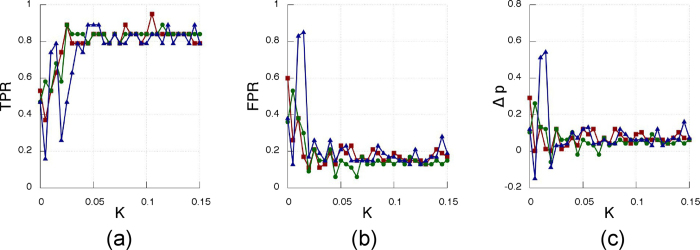
As in Fig. 9 but for the phases computed via a Hilbert transformation.

**Figure 11:**
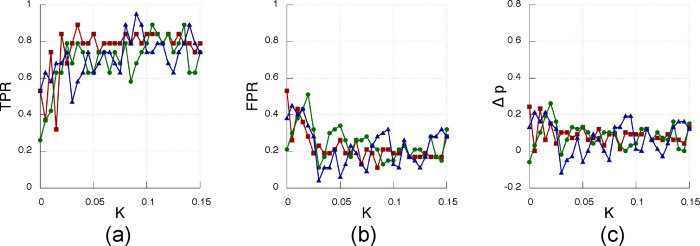
Same as Fig. 10 **but for the instantaneous frequencies.**

The results of the inference method applied to the data generated from the Rössler electronic oscillators are plotted in Fig. 9 (experimentally recorded time-series of the oscillator’s variable *x*_*i*_(t) with *i* = 1…12, Fig. 10 (oscillator’s phases computed via the Hilbert transform) and Fig. 11 (oscillator’sinstantaneous frequencies computed from their phases). As we can see, the three SSMs behave very similar when compared among them, but have different performance depending on the observable variable.

Figure 9 shows the results when the observable is *x*(t). We can see how, after a critical coupling around *K*_*c*_ = 0.002, the TPR and FPR quickly converge to 0.9 and 0.15 respectively, leading to a good network reconstruction, although it is not possible to fully retrieve the structural network, unlike in the Kuramoto case.

When using the phases (Fig. 10) or the instantaneous frequencies (Fig. 11) to infer the network, the performance is slightly lower than the one obtained with *x*(t). Table 2 summarizes the best performance for all possible combinations of SSMs and observables.

Since the dynamical properties of the three Rössler oscillators components are different – especially those of the *z* variable – we speculate that the algorithm performance could be different when applied to *y* or *z* variables, although we cannot check this it because for technical limitations it was possible to record only the x_*i*_ time-series.

## Conclusions and Discussion

We have analysed the relation between inferred functional networks and the underlying structural networks in two different systems, a simulated set of Kuramoto oscillators and an experimental set of Rössler chaotic electronic circuits. We focused in the dependence on the strength of the interaction among the system components, in the similarity measure used, and in the type of variable analysed.

For the first time we have contrasted nine inference methods (using three types of variables - amplitude, phase and frequency, and three similarity measures - cross correlation, mutual information, and ordinal mutual information), and we presented a detailed comparison of their performance using both synthetic and empirical data.

The first important result that we show here is that it is indeed possible to find regimes of the dynamics in which the functional connectivity of the system perfectly mimics the structural one (Kuramoto oscillators), or in which the former is a very good approximation of the latter (Rössler oscillators).

A second conclusion of our study is that functional networks obtained from thresholding the cross-correlation matrix perform better with respect to the two other non-linear measures used (the mutual information computed from histograms of values and the mutual information computed from histograms of ordinal patterns). We can see that, for Kuramoto oscillators, CC gives better results than both, MI and MIOP, thus the coarse grained covariance matrix, in a range of *K* values, is a good approximation of the adjacency matrix of the network. The fact that the linear measure, CC performs much better than the non-linear one is explainable at least for the high values of *K*. In fact, for high *K* the dynamics is almost synchronised, it means that the interaction term can be considered approximately linear. In fact, if K is high enough such that *R(t)* ∼ 1, we have *θ*_*i*_ ≃ *θ*_*j≠i*_ and the Kuramoto equation reads
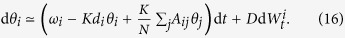
where *d*_*i*_ is the fraction of nodes connected with node *i*. It is clear that in general, in a linear system, the covariance matrix contains a great amount of information regarding the system.

Also for the frequencies the CC performs better than the non-linear measures and the reason for this can be found again in eq. (16). When we correlate two frequencies *v*, we multiply directly the interaction terms in the r. h. s. of eq. (16), where the connectivity appears in a linear way. In this way the covariance matrix reflects directly the adjacency one and we have an accurate reconstruction. Moreover, we shown that the CC for the frequencies develops a gap between the values of connected and unconnected pair of nodes, and this is in ultimate analysis the reason for which the thresholding procedure retrieves the network without any mistake.

We also shown that, for low values of *K* the FPR is higher than for high values of *K*, an effect that can be understood in terms of the results presented in the previous sections about the possibility of high cross-correlation values for the phases of uncoupled oscillators.

A third conclusion of our study refers to the influence of inferring the functional network by means of an indirect variable. Specifically, we found that in the Kuramoto oscillators the use of an indirect variable, such as the *Y* projection, can lead to errors in the network reconstruction, although the density of the inferred network is consistent with that of the structural network. On the other hand the use of the frequencies led to a better result than the one sing the phases. For the Rössles, instead, the indirect and direct variables give similar results.

We have also shown that the system’s size and its connectivity density can compromise the network reconstruction. When exporting the methodology to large networks, such as climate networks, we do not expect the network density to be a serious issue, since these networks are usually sparse; however, their large size could result in poor performance.

The problem of extending these results to real-world systems, however, remains an issue. We stress the fact that the use of a uniform, global threshold of the SSMs values could be a drawback if exporting the methodology to systems in which the dynamical units (or the strengths of their interaction) are heterogeneous; however, a possible way to overcome this drawback is to use a local threshold instead of a global one, i.e., a threshold defined for each pair of time-series. Namely, for each pair of time-series, a certain number of surrogate time-series can be built (for example with bootstrap techniques) and a distribution of surrogate SSMs for each pair of time-series is obtained. The threshold then can be defined locally as a certain percentile of the surrogate distribution. Therefore, it will be interesting for future work to use local thresholds to study the reconstruction of the physical connectivity when the system displays heterogeneity (in the dynamical units or in their interactions) and to develop a reliable, unambiguous way to determinate the threshold, either local or global.

For future work it will also be interesting to analyse how the network topology affects the performance of the inference method. We suppose that in the scale free case, the architecture detection could be more difficult. The reason is that, in our study, we are using a method that assumes implicitly an homogeneous structural architecture – in fact, as stated before, we used a constant threshold to prune the SSMs matrix – an hypothesis clearly violated in a scale free network, which presents highly connected nodes as well as poorly connected ones. In small world networks, instead, is harder to guess which might be the performance of the functional network approach. For sure, in the Kuramoto case, since we shown that the good performance of the algorithm relies in the quasi-synchronisation of the nodes, we expect to experience good reconstruction for *K* values bigger than the ones presented here because it is well known that random networks are more easily synchronisable than small-world ones.

It would also be very interesting to consider the possibility of improving the performance of the inference methodology by using a “multi-layer” approach, that is by considering the functional networks obtained from the intersection of those obtained from different variables.

Also link dependence on time is an issue that deserves attention, especially in the framework of climate and neuronal dynamics, where climate tipping points or neuronal plasticity change the structural connectivity in time; it would be interesting to understand how this behaviour if reflected in the functional connectivity, and which property of the structural one could be inferred using the associated functional network.

## Additional Information

**How to cite this article**: Tirabassi, G. *et al.* Inferring the connectivity of coupled oscillators from time-series statistical similarity analysis. *Sci. Rep.* **5**, 10829; doi: 10.1038/srep10829 (2015).

## Supplementary Material

Supplementary Information

## Figures and Tables

**Table 1:** 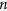
 -values for all the possible combinations of observables and SSMs in the Kuramoto case.

**Observable**	**CC**	**MI**	**MIOP**
Phases	0.5	0.5	1.0
Frequency	0.5	0.5	0.5
Projections	1.0	1.0	1.0

**Table 2:** Best performance for a network of 12 randomly coupled Kuramoto oscillators for 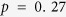 and 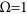 . The best true positive fraction (BTP) and the associated best false positive fraction (BFP) are chosen as the TPR and the FPR closest to the (1,0) corner in the (TPR,FPR) plane.

**Observable**	**BTP**	**BFP**	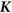	**SSM**
Phases	0.992	0.104	14.0	CC
Frequency	1.000	0.000	20.8	CC
Projections	0.583	0.251	4.8	MIOP

The associated 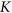 value is reported togehter with the corresponding SSM to which these best results are associated.

**Table 3 t3:** Best performance results of network inference of 12 randomly coupled Rössler chaotic oscillators.

**Observable**	**BTP**	**BFP**	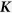	**SSM**
Phases	0.950	0.170	0.110	CC
Frequency	0.89	0.19	0.040	CC
Projections	0.890	0.130	0.025	MI

The best true positive fraction (BTP) and the associated best false positive fraction (BFP) are chosen as the TPR and FPR closest to the (1,0) corner in the (TPR,FPR) plane. The associated 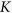 value is reported together with the corresponding SSM value.
