# Supplementary material for: Inferring the connectivity of coupled oscillators from time-series statistical similarity analysis
Source: Sci Rep. 2015 Jun 4;5:10829. doi: 10.1038/srep10829 (PMC4455306; doi:10.1038/srep10829)
Supplement: Supplementary Information [file srep10829-s1.pdf]

Supplementary Information:

Inferring the connectivity of coupled oscillators from time-series statistical similarity analysis

Giulio Tirabassi[1*], Ricardo Sevilla-Escoboza[2], Javier M. Buldú[3,4], Cristina Masoller[1]
1 Departament de Fisica i Enginyeria Nuclear, Universitat Politécnica de Catalunya, 08222 Terrassa, Barcelona, Spain
2 Center for Biomedical Technology, Technical University of Madrid, Pozuelo de Alarcón, 28223 Madrid, Spain
3 Centro Universitario de los Lagos, Universidad de Guadalajara, Lagos de Moreno, Jalisco 47460, Mexico
4 Complex Systems Group, Universidad Rey Juan Carlos, 28933 Móstoles, Madrid, Spain

1 Experimental electronic implementation of Rössler oscillators

The experiment consists of twelve electronic circuits, each of them being a piecewise Rössler-like system [1–3] operating in the chaotic regime, whose dynamics is given by:

$$\dot{x}_i = -\alpha \left(\Gamma x_i + \beta y_i + \lambda z_i - K\psi \sum_{j=1}^{N} A_{ij}(x_j - x_i) \right) \quad (1)$$

$$\dot{y}_i = -\alpha \left(-x_1 + \upsilon y_1 \right) \quad (2)$$

$$\dot{z}_i = -\alpha \left(-g(x_1) + z_1 \right) \quad (3)$$

where the piecewise part is:

$$g(x) = \begin{Bmatrix} 0 & if \quad x_i \leq 3 \\ \mu(x_i - 3) & if \quad x_i > 3 \end{Bmatrix}$$

x, y and z are the oscillator state variables, K is the coupling strength, $g(x)$ is the piecewise linear function and A_{ij} is the adjacency matrix containing the structure of the network. Here, $\alpha = 10^4\,\mathrm{s}^{-1}$ is a time factor, and the other parameters are: $\Gamma = 0.05$, $\beta = 0.5$, $\lambda = 1$, $\psi = 1$ $\mu = 15$ and $\upsilon = b - 0.02$. We choose $b = 1.66$ to have chaotic dynamics in the absence of coupling.

It is possible to translate the Rössler-like equations into the electronic circuit shown in Fig. 1 [4–8], which leads to the following equations of the system:

$$\dot{x}_i = -\frac{1}{R_1C_1}\left(x_i + \frac{R_1}{R_2}y_i + \frac{R_1}{R_4}z_i - K\frac{R_1}{R_x}\sum_{j=1}^{N}A_{ij}\left[x_j - x_i\right]\right) \quad (4)$$

$$\dot{y}_i = -\frac{1}{R_6C_2}\left(-\frac{R_6R_8}{R_9R_7}x_i + \left[1 - \frac{R_6R_8}{R_CR_7}\right]y_i\right) \quad (5)$$

$$\dot{z}_i = -\frac{1}{R_{10}C_3}\left(-\frac{R_{10}}{R_{11}}G_{x_i} + z_i\right) \quad (6)$$

$$(7)$$

where the piecewise part is:

$$g(x) = \begin{Bmatrix} 0 & if \quad x_i \leq I_d + I_d\frac{R_{14}}{R_{13}} + V_{ee}\frac{R_{14}}{R_{13}} \\ \frac{R_{12}}{R_{14}}x_i - V_{ee}\frac{R_{12}}{R_{13}} - I_d\left(\frac{R_{12}}{R_{14}} + \frac{R_{12}}{R_{13}}\right) & if \quad x_i > I_d + I_d\frac{R_{14}}{R_{13}} + V_{ee}\frac{R_{14}}{R_{13}} \end{Bmatrix}$$

Table 1 summarizes the values of all components used for the contraction of the Rössler-like system.

$C_1 = 1nF$	$C_2 = 1nF$	$C_3 = 1nF$	
$R_1 = 2M\Omega$	$R_2 = 200k\Omega$	$R_3 = 10k\Omega$	$R_4 = 100k\Omega$
$R_5 = 50k\Omega$	$R_6 = 5M\Omega$	$R_7 = 100k\Omega$	$R_8 = 10k\Omega$
$R_9 = 10k\Omega$	$R_{10} = 100k\Omega$	$R_{11} = 100k\Omega$	$R_{12} = 150k\Omega$
$R_{13} = 68k\Omega$	$R_{14} = 10k\Omega$	$R_{15} = 100k\Omega$	$R_c = R_3 + R_5$
$I_d = 0.7$	$V_{ee} = 15$		

Table 1. Values of the components used for the construction of the Rössler-like circuit described by Eqs. 4

Finally, Fig. 2 corresponds to the electronic circuit introducing diffusive coupling between nodes.

2 Analysis of the distribution of Cross Correlation values for two uncoupled Kuramoto oscillators.

In Fig. 3 of the main manuscript we reported that uncoupled Kuramoto oscillators can display arbitrary large values of cross correlation among them, independently of the length of the time-series. This is doubtlessly a counterintuitive feature, although we can prove that it has a solid mathematical reason.

Namely, what we are plotting in Fig. 3 is the maximum value over an ensemble of 120 possible correlations among the time series of uncoupled Kuramoto oscillators. The equations for this system can be written in the form

$$\mathrm{d}\theta_i = \omega_i \,\mathrm{d}t + D\,\mathrm{d}W_i^t, \tag{8}$$

and, since we processes the series removing a linear trend and normalizing respect to the variance, it can be reformulated as an autoregressive process of order 1 (AR(1)) and unitary memory:

$$\theta_i^{t+1} = \theta_i^t + \epsilon_i^t \tag{9}$$

where ϵ is a gaussian innovation with mean 0 and variance 1.

The correlation among two independent AR(1) processes can be proven to be equal to the correlation among the first stochastic elements of the two time-series, namely $r_W = \langle \epsilon_i^1 \epsilon_j^1 \rangle$ [9]. This correlation, of course, cannot be dependent of the length of the time-series since it is relative to the first step. (This explains why we detect an independence of the maximum value respect to the integration time.

The reason why the maximum can reach very high values can also be understood: the distribution of the AR(1) correlation can be transformed into a normal one with the so-called Fisher's transformation: $Z = \tanh^{-1}(r_W)$. This transformation is monotonous, so the maximum values of Z correspond univocally to the maximum values of r_W. Now, as stated in the caption of the figure, we are considering the maximum over $k = 120$ independent realizations, that is the maximum over 120 independent values of r_W i.e. of Z. We call this variable $x = \max(Z)$. The cumulative distribution of such a maximum, $F(x)_k$, can be proven to be given by the following relation: $F_k = [F_1]^k$ that, in our case, is the k-th power of the error function plus a constant. In fact, the probability that the maximum over k independent values is less than a certain x is equivalent to the probability that all the k values are below x, for the definition of maximum; this simply means $F_k = [F_1]^k$.

It has to be noted that for k sufficiently large the distribution F_k converges to a limit function referred as General Extreme Value (GEV) distribution, that is the analogous of the normal distribution of the central limit theorem for extreme values, i. e., the convergence of the x PDF to this function is guaranteed independently of the form of the Z PDF.

Now that we have the cumulative distribution of the variable $x = \max(Z)$ it is possible to compute its expectation as

$$m = \int_{-\infty}^{+\infty} x \, \frac{\mathrm{d}F_k}{\mathrm{d}x} \, \mathrm{d}x \tag{10}$$

This integral can be numerically estimated as $m \sim 2.57$ for $k = 120$ that lead, with an inverse Fisher's transformation, to the expected maximum correlation of $r_m \sim 0.988$. Estimating also the variance give use the one sigma interval [0.973; 0.995] for the expected value r_m, corresponding to a $\sim 70\%$ confidence interval.

Regarding the Y-projection case, instead, we cannot say much, since the nonlinear transformation to the AR(1) processes impel to compute neither the correlation explicitly nor the moments of the distribution via the generatrix function in a simple way.

For the frequencies-CC maximum values, instead, we have a decrease of the maximum with increasing length of the time-series, T, as $1/T$. This can be easily explained assuming that the maximum values scale with the length of the time-series together with the variance, since the distribution moments of the correlation are computable using the associated moment-generating function, and it can be seen that the variance indeed scales as $1/T$. In fact, from equation 9 it is possible to see that the cross correlation for two frequencies time-series can be written as

$$CC_{ij} = \frac{1}{T} \sum_{t}^{T} \epsilon_i^t \, \epsilon_j^t. \tag{11}$$

We call the product of two normal independent variable $z^t = \epsilon_i^t \, \epsilon_j^t$. In can be proven that this variable follows a certain distribution with an associated characteristic function $\phi_z(u) = (1+u^2)^{-1/2}$. Thus, being z^t independent variables, the characteristic function of CC_{ij} can be written as

$$\phi_{CC}(u) = \prod_{t}^{T} \phi_{z^t} \left(\frac{u}{T} \right) = \left[\phi_z \left(\frac{u}{T} \right) \right]^T. \tag{12}$$

Deriving twice and evaluating for $u = 0$ we obtain the variance of CC, namely

$$\mathrm{Var}[CC] = \frac{1}{T}. \tag{13}$$

References

1. Carroll TL, Pecora LM (1995) Nonlinear dynamics in circuits. World Scientific.

2. Pisarchik AN, Jaimes-Reátegui R, García-López JH (2008) Synchronization of coupled bistable chaotic systems: experimental study. Philosophical Transactions of the Royal Society A: Mathematical, Physical and Engineering Sciences 366: 459–473.

3. Pisarchik A, Jaimes-Reátegui R, Villalobos-Salazar J, Garcia-Lopez J, Boccaletti S (2006) Synchronization of chaotic systems with coexisting attractors. Physical Review Letters 96: 244102–244102.

4. Pecora, L. M., Carroll, T. L. (1998). Master stability functions for synchronized coupled systems. Phys. Rev. Lett. 80(10), 2109.

5. Heagy, J. F., Carroll, T. L., Pecora, L. M. (1994). Synchronous chaos in coupled oscillator systems. Phys. Rev. E, 50(3), 1874.

6. Fink, K. S., Johnson, G., Carroll, T., Mar, D., Pecora, L. (2000). Three coupled oscillators as a universal probe of synchronization stability in coupled oscillator arrays. Physical Review E, 61(5), 5080.

7. Heagy, J. F., Carroll, T. L., Pecora, L. M. (1995). Desynchronization by periodic orbits. Phys. Rev. E, 52(2), R1253.

8. Boccaletti, S., Valladares, D. L., Pecora, L. M., Geffert, H. P., Carroll, T. (2002). Reconstructing embedding spaces of coupled dynamical systems from multivariate data. Physical Review E, 65(3), 035204.

9. Mudelsee M, Climate time-series analysis, Springer, *2nd ed.* (2014), pag. 276.

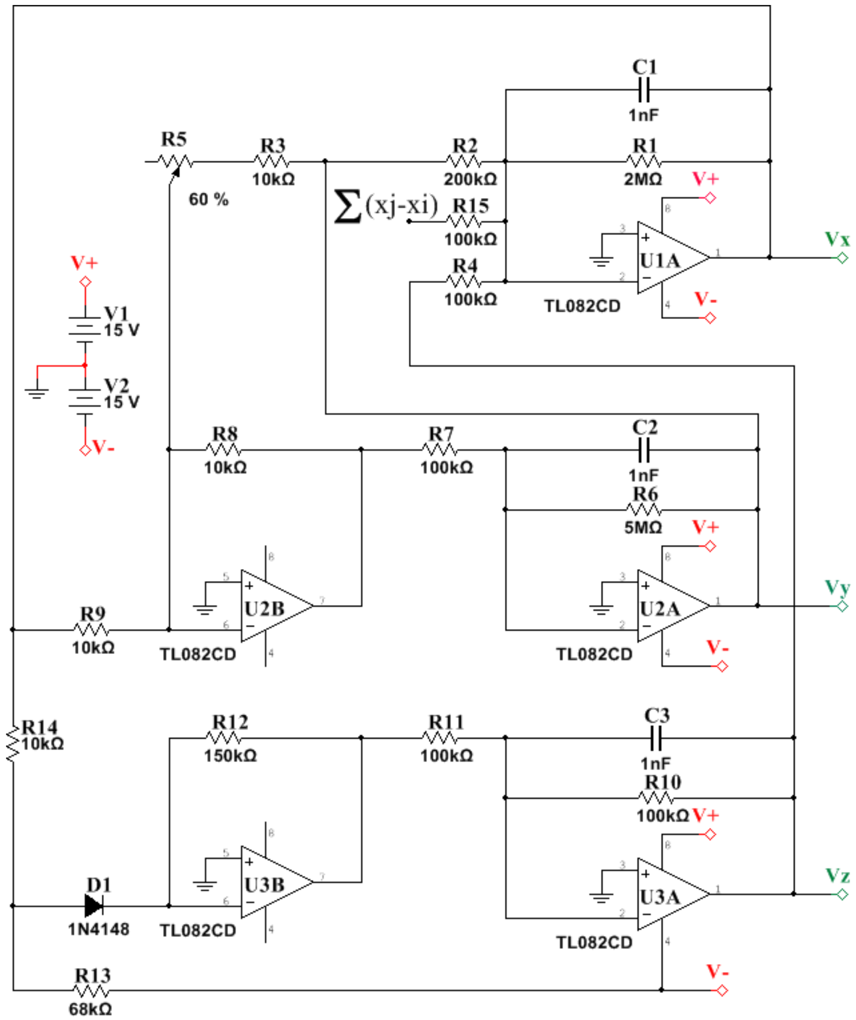

Figure 1. Electronic implementation of a Rössler-like electronic circuit. The values of the parameters of the electronic components are summarized in Tab. 1. The term $\sum(x_j - x_i)$ accounts for the diffusive coupling between units, and the corresponding electronic circuit is shown in Fig. 2.

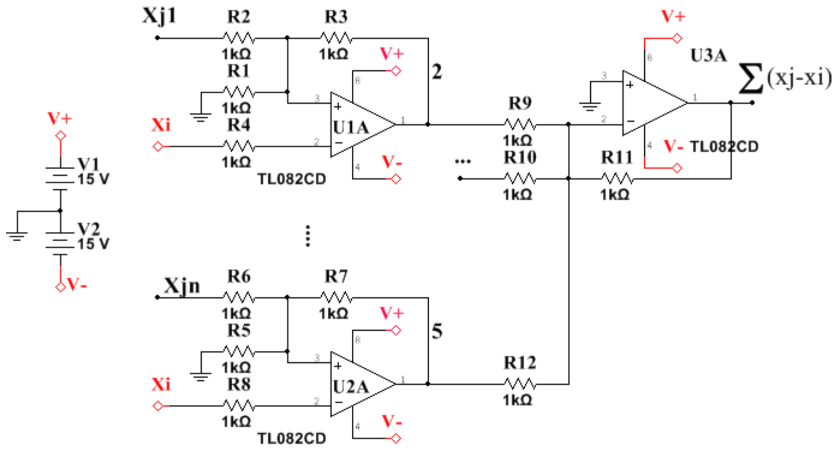

Figure 2. Electronic implementation of the diffusive coupling between a Rössler-like system and all of its n neighbours. Each branch of the circuit accounts for the difference between oscillator i and j, being a total of n branches. Finally a voltage adder joins the output of each branch.
